# Role of Group 1 CD1-Restricted T Cells in Infectious Disease

**DOI:** 10.3389/fimmu.2015.00337

**Published:** 2015-06-29

**Authors:** Sarah Siddiqui, Lavanya Visvabharathy, Chyung-Ru Wang

**Affiliations:** ^1^Department of Microbiology and Immunology, Northwestern University Feinberg School of Medicine, Chicago, IL, USA

**Keywords:** *Mycobacterium tuberculosis*, CD1, antigen presentation, T cells, NKT cells, animal models

## Abstract

The evolutionarily conserved CD1 family of antigen-presenting molecules presents lipid antigens rather than peptide antigens to T cells. CD1 molecules, unlike classical MHC molecules, display limited polymorphism, making CD1-restricted lipid antigens attractive vaccine targets that could be recognized in a genetically diverse human population. Group 1 CD1 (CD1a, CD1b, and CD1c)-restricted T cells have been implicated to play critical roles in a variety of autoimmune and infectious diseases. In this review, we summarize current knowledge and recent discoveries on the development of group 1 CD1-restricted T cells and their function in different infection models. In particular, we focus on (1) newly identified microbial and self-lipid antigens, (2) kinetics, phenotype, and unique properties of group 1 CD1-restricted T cells during infection, and (3) the similarities of group 1 CD1-restricted T cells to the closely related group 2 CD1-restricted T cells.

## Introduction

The *CD1* gene family encodes several MHC class I-like antigen-presenting molecules, which are specialized to present lipid antigens to T cells. The lipid antigens presented by CD1 include a diverse array of lipids/glycolipids, ranging from foreign lipids unique to specific microorganisms to common mammalian self-lipids. Three major groups of CD1 isoforms have been identified in humans: group 1 CD1 (CD1a, CD1b, and CD1c), group 2 CD1 (CD1d), and group 3 CD1 (CD1e) (1). Although homologs exist in the guinea pigs and other vertebrates, mice, and rats do not express group 1 CD1 (2, 3), making the *in vivo* functional study of group 1 CD1-restricted T cells difficult until the recent development of small animal models (4–6). This review will outline group 1 CD1 antigen binding and presentation, newly identified group 1 CD1-restricted self- and microbial lipid antigens, and the interaction of group 1 CD1-expressing antigen-presenting cells (APCs) with their corresponding T cell subsets. Additionally, the development and function of group 1 CD1-restricted T cells will be discussed, with special emphasis placed on small animal models, dynamics of these T cells during *Mycobacterium tuberculosis* (*Mtb*) infection, and the contribution of microbial antigen-specific and autoreactive group 1 CD1-restricted T cells to host defense against infection. Finally, the expansion of our knowledge of group 1 CD1-restricted T cell responses allows for their comparison with a more well-studied and related subset of T cells, the group 2 CD1-restricted NKT cells. The last section will focus on comparing and contrasting the properties of group 1 vs. group 2 CD1-restricted T cells.

## Expression of Group 1 CD1 Molecules

Unlike MHC class Ia, which is expressed on all nucleated cells, the expression of group 1 CD1 is mainly limited to APCs and double-positive (CD4^+^CD8^+^) cortical thymocytes (1). CD1a molecules are highly expressed on skin resident DCs, or Langerhans cells (7). CD1b is most highly expressed on a subset of migrating lymph dendritic cells and myeloid-derived dendritic cells (8). CD1c is the most ubiquitously expressed group 1 CD1 molecule, being found on monocyte-derived DCs, B cells, and Langerhans cells under steady-state conditions (9, 10). CD1e is the only CD1 isoform that is not expressed on the surface of APCs (11). In immature DCs, CD1e is mainly localized in the Golgi apparatus and in mature DCs, it is detected in late endosomes, where it is cleaved into a functional soluble form (12). CD1e is thought to facilitate the processing and presentation of certain lipid antigens presented by CD1b (13).

Group 1 CD1 expression can also be regulated by various cytokines. Treatment of activated monocytes with GM-CSF and IL-4, which leads to dendritic cell differentiation, results in increased levels of group 1 CD1 expression (14). Group 1 CD1 expression is also altered in different disease models, implying a role for group 1 CD1-restricted T cells in the pathology and/or repression of the disease. Patients with the tuberculoid form of leprosy, a clinical pattern associated with active cellular immunity toward *Mycobacterium leprae*, show induction of CD1a, CD1b, and CD1c on DCs in dermal granulomas (15). Increased CD1b and CD1c expression has also been reported in the human skin upon *Borrelia burgdorferi* infection (16). CD1a expression on Langerhans cells has been correlated with a variety of autoimmune skin diseases, including atopic eczema (7, 17, 18) and psoriasis (19, 20). In addition, some leukemias and lymphomas express one or all group 1 CD1 molecules (21). One recent study showed that CD1c^+^ B cell leukemia precursors are efficiently targeted and lysed by CD1c autoreactive T cells, highlighting a role of group 1 CD1-restrcited T cells in anti-tumor immunity (22).

## Binding and Presentation of Lipid Antigens by Group 1 CD1 Molecules

Group 1 CD1 isoforms have structurally diverse antigen-binding grooves that allow them to bind very different lipid classes. Group 1 CD1 isoforms also differ in their stability when lipids are bound in the binding groove vs. when the groove lies empty, which is consistent with their intracellular trafficking patterns and consequently the types of lipids they are exposed to (23, 24). CD1b and CD1c molecules traffic through and load lipids in the late endosome and the lysosome, and thus, are exposed to a low pH that may facilitate lipid exchange (25). However, CD1a molecules are unique in that under steady-state conditions, they do not accumulate or traffic through the late endosomes or lysosomes, and thus, are not exposed to the same low pH conditions as CD1b and -c molecules (26). Instead, modeling simulations suggest that CD1a molecules can be stably expressed on the cell surface in the absence of loaded lipid antigens (27), and other studies have shown that the CD1a binding groove can be stabilized by exogenously added lipids after being expressed on the cell surface (28).

Group 1 CD1 molecules present both microbial and self-lipid antigens to T cells. Historically, microbial antigens that were found to bind to group 1 CD1 molecules were derived from the cell wall of *Mycobacteria* species. Below, we will describe how the various group 1 CD1 molecules interact with different types of lipids, and what some of the functional consequences for T cell activation might be.

### Lipid antigen presentation by CD1a

CD1a molecules can bind the mycobacterial lipopeptide didehydroxymycobactin (DDM), a biosynthetic precursor of the iron-chelating siderophore mycobactin, which is essential for *Mtb* growth within macrophages (29). CD1a presentation of DDM and subsequent TCR recognition of antigen have features reminiscent of both CD1 and MHC-like binding modes in that the CD1a-restricted TCR is capable of responding in an amino-acid sequence-specific manner to the solvent-exposed portion of the DDM lipopeptide (30). DDM-specific CD1a-restricted T cells were found to secrete varying levels of IL-2 in response to different synthetic DDM analogs, with the largest amount of IL-2 being produced when the DDM antigen was in its native state (31). This showed that CD1a-restricted T cells could specifically respond to the structural components of an antigen that are responsible for virulence (31).

The crystal structure of CD1a–DDM complex showed that A′ pocket of CD1a antigen-binding groove binds alkyl chains of discrete length, while the F′ pocket is less rigid and allows for different chemical head groups of lipid antigens to protrude from the groove (30). The crystal structure of CD1a with the bound self-antigen sulfatide provides another example of this type of binding mode wherein the sphingosine fits into the A′ pocket and the polar headgroup of sulfatide protrudes from the middle of the groove, allowing for optimal TCR recognition (32). However, despite the body of work showing that CD1a lipid antigens generally contain protruding polar headgroups and hydrophobic tails, a recent study showed that CD1a molecules could also bind and present “headless” lipid antigens to T cells (33). In fact, synthetic squalene, a lipid containing no headgroup, was able to activate the BC2 CD1a-restricted T cell line to the same extent as what was seen from challenge with sebaceous gland extract containing a mixture of CD1a autoantigens (33). This suggests that CD1a has the ability to recognize “headless” lipid antigens that lack a polar moiety available to be recognized by the TCR. A recent finding by Rossjohn’s group provided a potential explanation for this (34). This study first solved the crystal structure of a TCR bound to CD1a and mechanistically showed that the TCR can directly contact CD1a in the absence of lipid interaction. This interaction is sufficient for T cell activation. However, if CD1a is loaded with non-permissive lipids, there is a disruption of the TCR–CD1a docking site (34). Therefore, CD1a autoreactivity can result from direct contact of the TCR with CD1a, but only when the surface of CD1a is not disrupted by non-permissive ligands. Furthermore, a recent study showed that bee venom-derived phospholipase 2 could activate CD1a-autoreactive T cells through the generation of small neo-antigens, such as free fatty acids and lysophopholipids, from common phosphodiacyl glycerides (35). This finding provides a potential mechanism underlying phospholipase-dependent inflammatory skin disease.

### Lipid antigen presentation by CD1b

CD1b has the largest antigen-binding groove among the CD1 proteins described to date. As such, CD1b binds and presents the most diverse array of mycobacterial lipids, which include mycolic acid (36), glucose monomycolate (GMM) (37), glycerol monomycolate (38), phosphatidylinositol mannosides (PIMs) (39, 40), lipoarabinomannans (LAM) (41–43), and sulfoglycolipids (44). The CD1b binding groove is composed of four interconnected pockets, which enable binding of the long hydrophobic tails of lipid antigens (45). The structure of CD1b–GMM complex showed that the A′, C′, F′, and T′ pockets in the CD1b binding groove form a deep “maze” that allows them to present large mycobacterial lipid antigens to T cells, and the close association of lipid ligands with CD1b molecules is suggestive of this complex having a long half-life (45). These studies also show that the pleiotropic binding of structurally diverse lipids to CD1b molecules can occur due to critical amino-acid residues in the CD1b binding groove having the ability to change their conformation to induce a tighter fit for different lipids (45, 46). However, the finding that CD1b molecules bind and are stabilized by such large microbial lipid ligands led to the question of how unloaded CD1b molecules retain their structure in the absence of endosomally loaded lipid. Operating from the hypothesis that endogenous self-lipids could act as stabilizers of the Ag-binding groove for CD1b in the absence of foreign ligands, Garcia-Alles et al. showed that CD1b associates with phosphatidylcholine (PC) and a long 40+ carbon spacer molecule prior to endosomal lipid loading (47). The combined number of carbons shared between PC and the spacer molecule is greater than the C65–C70 capacity of the CD1b binding groove, but this “overloading” of the groove may actually facilitate lipid exchange in the endolysosome (47).

Mycolic acids were the first CD1b-restricted lipids identified (36). Mycolic acids are the major *Mtb* cell wall lipid components and are crucial for *Mtb* virulence (48). Studies in untreated human *Mtb* patients showed that many patients contained IFN-γ producing, mycolic acid-specific CD1b-restricted T cells in their PBMCs (49), suggesting that mycolic acids are immunodominant CD1b-restricted antigens relevant during *Mtb* infection. While free mycolic acids can activate some CD1b-restricted T cells; one recent study suggested that mycolic acids may act as scaffolds for other lipid antigens, with the tight association of the long hydrophobic tails in the CD1b binding pockets leading to proper positioning of various lipid antigenic headgroup for TCR recognition (38).

Diacylated sulfoglycolipids (Ac_2_SGL) are produced by virulent *Mtb* strains upon infection of the human host (44, 50). Studies using human CD1b-restricted T cell lines specific for Ac_2_SGL showed that the aliphatic tail region is responsible for anchoring the molecule deep in the CD1b antigen-binding groove, while the trehalose headgroup is responsible for TCR recognition (50). However, the antigen specificity of particular T cell lines could be influenced by the length and isomerization of the hydrophobic tail region of Ac_2_SGL (50). This corroborates the study described above, which showed that the long hydrophobic lipid tails of CD1b lipid antigens, including Ac_2_SGLs, can act to position the antigenic headgroups optimally for TCR recognition (38, 47).

*Mycobacterium tuberculosis* PIMs and their polyglycosylated extensions lipomannans (LM) and LAM (51) are components of the *Mtb* cell envelope (52). Many forms of LM and LAM are presented by CD1b (42, 43). Biochemical and functional characterization of structurally diverse forms of LM showed that the pathogenic *Mtb* strain H37RV contained LM moieties with longer and more branched mannan polymer domains than those from the avirulent strain *Mycobacterium smegmatis* (43). Thus, it is possible that virulent *Mtb* strains evolved to use their LM lipids as immune evasion factors, as CD1b–LM complexes from H37Rv were less potent activators of CD1b-restricted T cell responses than those from *M. smegmatis* (43).

### Lipid antigen presentation by CD1c

The only known *Mtb* antigen presented by CD1c is phosphomycoketide (PM), a lipid that contains a single fully saturated alkyl chain with methyl branches at every fourth carbon (53, 54). The production of *Mtb* mannose-1-β-phosphomycoketide (MPM) occurs via enzymatic catalysis by Pks12, which is only found in slow-growing pathogenic strains of *Mtb* (55), suggesting that MPM is a potential virulence factor in *Mtb* pathogenesis. Recent studies showed that PM, a putative biosynthetic precursor for MPM can also bind to CD1c and activate CD1c-restricted T cells (56).

CD1c molecules have a unique antigen-binding mode that allows for presentation of diverse antigens, such as highly branched lipid species to T cells (57). Whereas lipid antigens bind in both the A′ and F′ pockets in CD1a and CD1b molecules (32, 45), MPM binds only to the A′ pocket in CD1c molecules, allowing for the branched side chains to protrude out from the groove (57). Like CD1a, CD1c can also present lipopeptide antigens to T cells (58). In addition, CD1c has been shown to bind sulfatide and other diacylated lipids similar to CD1a and CD1b (50, 59), suggesting that the CD1c binding groove has evolved to present a diverse array of lipid species.

A recent paper also helped shed light on a potential role for autoreactive CD1c-restricted T cells (22). Autoreactive CD1c-restricted T cells were found to recognize methyl-lysophosphotidic acid, a novel class of self-lipids, which accumulate in leukemia cells (22). While these autoreactive CD1c-restricted T cells poorly recognize non-tumor CD1c-expressing cells, they killed CD1c^+^ acute leukemia cells and protected immunodeficient mice against CD1c^+^ human leukemia cells (22). This study suggests that autoreactive CD1c-restricted T cells may play a role in anti-tumor immunity (22). However, their role in anti-microbial immunity remains unknown.

## Kinetics, Phenotype, and Unique Properties of Group 1 CD1-Restricted T Cells

Much of the information about group 1 CD1-restricted T cells comes from *in vitro* assays using human T cell clones. Recent discoveries in tetramer development and improved cell isolation technology have greatly enhanced the study of group 1 CD1 molecules in humans, though animal models are critical to fully understand the dynamic biology of group 1 CD1-restricted T cells. A recent study using rhesus macaques identified a GMM-specific T cell population in BCG-immunized monkeys (60). However, unlike humans, GMM-specific T cells were found to be CD1c restricted (61). Though interesting, following up on such findings in primates can be challenging due to cost constraints. As an alternative to studying non-human primates, small animal models using transgenic mice have proven invaluable for the study of group 1 CD1-restricted T cells. In the following sections, we will review recent discoveries about group 1 CD1-restricted T cells and the role they play in host immunity *in vivo*.

### Small animal models to study the *In vivo* function of group 1 CD1-restricted T cells

There have been a number of small animal models used to study the *in vivo* function of group 1 CD1-restricted T cell responses during *Mtb* infection, including the guinea pig model and humanized mouse model. Based on comparison with human CD1 isoforms, the nine guinea pig CD1 genes encode four CD1b-like, three CD1c-like, one CD1d-like, and one CD1e-like molecule (2, 62). Immunization of guinea pig with mycobacterial lipids has been shown to elicit antigen-specific proliferation and cytolytic capacity of group 1 CD1-restricted T cells (63). In addition, *Mtb* lipid-vaccinated guinea pigs exhibit reduced lung pathology after subsequent *Mtb* challenge (64). However, it has been difficult to show that group 1 CD1-restricted T cells mediate these protective effects due to limited experimental tools and reagents. Moreover, guinea pigs lack CD1a and have multiple isoforms of the CD1b and CD1c genes, suggesting that the group 1 CD1-restricted T cells response may be different in guinea pigs and humans.

A humanized mouse model developed by Gumperz’s group was observed to develop a functional CD1 compartment (6). This model involved grafting immunodeficient mice with human fetal thymus, liver, and CD34^+^ hematopoietic stem cells (6). While these mice were observed to express group 1 CD1 molecules, CD1a tissue expression patterns differed from humans (6). For instance, CD1a-expressing cells, which are normally abundant in human skin, are largely absent in humanized mice. It remains to be seen whether group 1 CD1-restrcited T cell responses can be elicited in these humanized mice upon immunization or during infection.

A transgenic mouse model that expresses the human group 1 *CD1* genes under their endogenous human promoters has been generated (4). Human group 1 CD1 transgenic (hCD1Tg) mice express CD1a, CD1b, and CD1c in a pattern similar to that seen in humans and support the development of group 1 CD1-restricted T cells (4). These T cells from hCD1Tg mice also share many phenotypic and functional characteristics with CD1-restricted T cells found in humans (4). These data demonstrate the viability of hCD1Tg mice as an animal model to study the *in vivo* function of group 1 CD1-restricted T cells. Indeed, both infection with *Mtb* and immunization with *Mtb* lipids elicit group 1 CD1-restricted T cell responses in hCD1Tg mice (4).

### Group 1 CD1-restricted T cell responses during *Mtb* infection

The presentation of mycobacterial lipid antigens by group 1 CD1 molecules has been well characterized. However, our understanding of the phenotype and function of responding T cells was mostly limited to *in vitro* assays with T cell clones until recently. Nevertheless, *in vitro* studies have demonstrated that group 1 CD1-restricted T cells are cytotoxic and produce IFN-γ and TNF-α upon encountering mycobacterial antigens (65, 66). Moreover, group 1 CD1-restricted *Mtb* lipid antigen-specific T cells are found in higher frequencies in individuals exposed to *Mtb* compared with a control population, suggesting that *Mtb*-specific CD1-restricted T cells are activated following infection with *Mtb* (38, 44, 65–67). The discovery of CD1-tetramer technology has made it easier to study T cell reactivity by enabling the direct isolation of antigen-specific T cells from the blood and tissue (56, 68). Moreover, the establishment of a group 1 CD1 transgenic mouse model has enabled the study of tissue distribution, activation kinetics, and phenotype of group 1 CD1-restricted T cells.

CD1a dextramers loaded with the mycobacterial lipopeptide DDM stained CD1a-restricted T cells in individuals with active TB and tuberculin-positive individuals, suggesting an expansion had occurred upon exposure to *Mtb* (69). CD1a/DDM dextramer^+^ T cells were found to be either CD4^+^ or CD8^+^, and produced IFN-γ and TNF-α in response to CD1a-expressing APCs treated with DDM. Studies with CD1b/GMM tetramers revealed the expansion of CD1b/GMM-specific T cells in individuals infected with *Mtb*, but not healthy individuals (68). These cells were found to be CD4^+^ TCRαβ^+^ and appeared to have a conserved TCR repertoire. CD1b/GMM-specific T cells were later separated into two types based on their avidity to CD1b/GMM. GMM-specific T cells with a high avidity to CD1b/GMM termed GEM (Germline-encoded, mycolyl-reactive) T cells expressed highly conserved TCRs composed of a TRAV1-2 (Vα 7.2) V segment rearranged with TRAJ9, with limited CDR3α diversity (70). GEM T cells were found at a very low frequency in *Mtb* naïve individuals. A second population with an intermediate avidity to CD1b/GMM was also observed and this population showed diverse TCR usage (71). Recently, Moody and colleagues described that CD1c-restricted T cells respond to glycosylated and unglycosylated forms of mycoketide presented by DCs or B cells (56). However, in cell-free systems, CD1c-restricted T cells responded only to unglycosylated forms of mycoketide (PM). Moreover, PM-loaded CD1c tetramers could detect antigen-specific T cells from PBMC of donors with latent TB. A comprehensive molecular analysis of TCR recognition of CD1c-mediated PM/MPM presentation revealed that some T cell clones recognize both PM and MPM while others are specific to either PM or MPM (72).

Studies infecting group 1 CD1-expressing hCD1Tg mice with BCG or *Mtb* revealed that the kinetics of group 1 CD1-restricted T cell responses were different from group 2 CD1d-restricted iNKT cells (4). Group 1 CD1-restricted T cells were detected at week 3 or 4 post infection, suggesting that they follow similar activation kinetics as peptide-specific conventional T cells. This study provided the first direct *in vivo* evidence that group 1 CD1-restricted T cell responses are induced by *Mtb* infection (4). Immunizing mice with BMDCs pulsed with *Mtb* lipid antigens followed by secondary challenge accelerated group 1 CD1-restricted T cell responses, indicating that group 1 CD1-restricted T cells can mount a memory response (4). Taken together, these studies support the potential use of group 1 CD1-restricted *Mtb* lipid antigens as components of subunit vaccines.

### Autoreactive group 1 CD1-restricted T cells

Early reports of T cell recognition of group 1 CD1 molecules suggested that many CD1-restricted T cells are autoreactive (73, 74). A recent analysis of T cell clones from human PBMC showed that CD1a self-reactive T cells are present at high frequencies in the blood of healthy individuals (75). CD1a-restricted autoreactive T cells expressed diverse TCRs and skin homing receptors CCR4, CCR6, CCR10, and secreted IL-22. Given the properties of CD1a-autoreactive T cells and the fact that CD1a recognizes skin lipid antigens and is highly expressed on Langerhans cells implies that these T cells play a role in dermal immunity (33, 75). Another study investigated that the frequency and effector phenotype of autoreactive group 1 CD1-restricted T cells in human blood and cord blood using C1R transfectants expressing CD1a, CD1b, and CD1c (76). They found that the frequencies of group 1 CD1 autoreactive T cells were in the range of 1/10–1/300 circulating T cells. CD1a- and CD1c-restricted T cells comprised the most abundant autoreactive T cells. These group 1 CD1 autoreactive T cells had a diverse phenotype and exhibited mostly Th1 and Th0 functional activities (76).

Most of the CTLs derived from hCD1Tg mice are autoreactive (4). While the endogenous lipid antigens that activate these CTLs were not identified in this study, these clones were found to lyse hCD1Tg DC and group 1 CD1-expressing cells from humans. This suggests that the self-antigens recognized by these CTLs are conserved between mice and humans. Autoreactive T cells can also be detected from *Mtb*-infected mice. A transgenic mouse model, HJ1Tg, expressing an autoreactive CD1b-restricted TCR has been generated to study the development and function of group 1 CD1 autoreactive T cells (5). Similar to CD1d-restricted iNKT cells, HJ1 T cells exhibited an activated phenotype (CD44^+^CD69^+^CD122^+^) and a subset of HJ1 T cells expressed NK1.1 and was enriched in the liver (5). This finding raises the possibility that autoreactive group 1 CD1-restricted T cells may also reside within the NKT cell compartment of the human liver. HJ1 T cells were cytolytic and secreted proinflammatory cytokines (IFN-γ, IL-17A, and TNF-α) in response to CD1b-expressing DCs. TLR agonists (Pam3Cys and LPS) enhanced the autoreactivity of HJ1 T cells by stimulating DCs to produce IL12/IL23. Furthermore, HJ1 T cells were activated early during *Listeria* infection and played an immunoprotective role (5). The potential role of group 1 CD1 autoreactive T cells in other infections remains to be defined.

### Developmental requirements of group 1 CD1-restricted T cells

The development of group 1 CD1-restricted T cells has not been well-studied due to a lack of a suitable small animal model, but some progress has been made in this regard. Studies using HJ1Tg mice showed that CD1b-expressing hematopoietic cells are necessary and sufficient to mediate the positive selection of HJ1 T cells (5). Cortical thymocytes are most likely the main cell type involved in the selection of HJ1 T cells as CD1b is not expressed on mature thymocytes. HJ1 T cells that developed in the hCD1Tg background expressed similar levels of the transcription factor PLZF as iNKT cells. PLZF is expressed by several non-conventional T cell subsets that display an activated phenotype and is responsible for their innate-like effector program (77–80). However, studies of group 1 CD1 autoreactive T cells in humans showed that autoreactive group 1 CD1-restricted T cells were present in both naive (CD45RA^+^) and effector/memory (CD45RO^+^) compartments (75, 76). It is unclear whether TCR–CD1 avidity and the nature of the selecting lipid antigen(s) may play a role in determining the phenotype of group 1 CD1 autoreactive T cells. Moreover, the developmental selection program for microbial lipid antigen-specific group 1 CD1-restricted T cells has not yet been investigated.

## Group 1 vs. Group 2 CD1-Restricted T Cells: Some Similarities, Many Differences

Group 1 and group 2 CD1 molecules share many similarities beyond the presentation of lipid antigens to T cells, including molecular assembly mechanisms and sharing self-lipid moieties that stabilize the CD1 binding groove in the absence of foreign antigen (81, 82). However, many differences also exist regarding the functional capacity of their cognate T cells and APC–T cell interactions. The following sections will focus on comparing and contrasting group 1 and group 2 CD1-restricted T cells in terms of TCR diversity, lipid recognition, cytokine production, and APC–T cell interactions. Table 1 highlights the properties of these two groups of CD1-restricted T cells.

**Table 1:** **Comparison of group 1 and group 2 CD1-restricted T cells**.

	Group 1 CD1-restricted T cells		Group 2 CD1-restricted T cells	
Types	Microbial antigen-specific	Autoreactive	Type I (iNKT)	Type II (vNKT)
Retricted by	CD1a, CD1b, CD1c		CD1d	
Antigens recognized	*Mtb* lipid antigens	CD1a – squalene, wax esters, triacylglycerides, sulfatide	α-GalCer analogs, glycosphingolipids, α-Gal-diacylglycerols, phospholipids, gangliosides, isoglobosides	β-GluCer, sulfatide, lysosulfatide, lysophospholipids
		CD1b – gangliosides, sulfatide		
		CD1c – methy-lysophosphatidic acid, sulfatide		
Antigen capture	CD1a – cell surface and early endosomes		Endosomes and lysosomes	
	CD1b – late endosomes and lysosomes			
	CD1c – endosomes			
Cell type that mediates selection	Unknown	Hematopoietic cells	Hematopoietic cells	
Subset	CD4^+^, CD8^+^, DN	CD4^+^, CD8^+^, DN	Human-CD4^+^, CD8^+^, DN	CD4^+^, CD8^+^, DN
			Mouse-CD4^+^, DN	
TCR usage	Diverse TCRαβ chains, Vα7.2-Jα9 (GEM T cells)	Diverse TCRαβ chains	Human – Vα24-	Diverse TCRαβ chains (mouse – Vα3.2, Vα8, Vβ8 bias)
			Jα18; Vβ11	
			Mouse – Vα14-	
			Jα18; Vβ8.2, Vβ7, Vβ2,	
Effector functions	Cytotoxic, produce IFNγ, TNFα	Cytotoxic, produce IFNγ, TNFα, IL-17	Cytotoxic, produce Th1, Th2, Th17 cytokines	Cytotoxic, produce Th1, Th2 cytokines

### TCR diversity in CD1-restricted T cells

CD1d-restricted NKT cells can be divided into two subsets based on TCR diversity (83). Type I NKT cells, also known as invariant NKT (iNKT) cells, express an invariant TCR α chain. Murine type I NKT cells express an invariant Vα14–Jα18 TCR α chain paired with a limited set of TCR β chains, including Vβ8.2, Vβ7, and Vβ2 (83). Like murine type I NKT cells, human type I NKT cells express the invariant Vα24–Jα18 TCR α chain paired with predominantly the Vβ11 chain (84). All type I NKT cells recognize the marine sponge-derived glycolipid, α-galactosylceramide (α-GalCer) (85). Other populations of CD1d-restricted NKT cells that respond to lipid antigens are broadly classified as type II NKT cells, which exhibit more TCR sequence diversity compared to type I NKT cells (86). Type II NKT cells do not respond to α-GalCer, and therefore, cannot be identified using α-GalCer/CD1d tetramers (86). Several studies have suggested that group 1 CD1-restricted T cells have diverse TCR usage (4, 33, 74, 76). However, CD1b-restricted GEM T cells were recently described to express highly conserved TCRs composed of a TRAV1-2V segment rearranged with TRAJ9, with limited CDR3α diversity (70).

### Lipid antigen recognition by group 1 and group 2 CD1-restricted T cells

Both group 1 and group 2 CD1 have been shown to present self-lipid antigens to CD1 autoreactive T cells. However, each member of the CD1 family binds distinct self-lipids. CD1d binds to sphingomyelin, gangliosides (e.g., GD3), globo/isoglobosides, phospholipids, plasmalogens, and lysophospholipids (82, 87–93). However, it remains unclear which of these lipids stimulate iNKT cells under physiological conditions and which contribute to the activation of iNKT cells during microbial infection (94). Gangliosides (e.g., GM1, GD1) were also described to bind to CD1b and activate CD1b-restricted T cells (95). Diacylglycerophosphocholines and diacylglycerophoshoinositol were shown to be bound by CD1c (87). However, it is unclear whether these self-lipids can be recognized by CD1c-restricted T cells. Self-lipids bound by CD1a molecules include squalene, wax esters, and triacylglycerides derived from the skin (33). Interestingly, sulfatide can bind to all group 1 and group 2 CD1 proteins (CD1a, CD1b, CD1c, CD1d) and activate CD1-restricted T cells (59, 96).

Most studies examining microbial antigen presentation by group 1 CD1 molecules have focused on *Mtb*, but CD1d-restricted iNKT cells have been shown to bind microbial antigens from a variety of pathogens. Both CD1d and CD1b molecules bind and present PIM moieties from *Mtb*, though they bind different isoforms (40, 97). In addition, diacylglycerol lipid species from pathogens including *B. burgdorferi* and *Streptococcus pneumoniae* have been identified as CD1d-restricted iNKT cell ligands, with physiologically relevant effects on T cell responses during infection (98, 99). No comparable diversity in microbial ligands has been shown to exist in group 1 CD1-mediated antigen presentation. However, the biochemical nature of the group 1 CD1 antigen-binding groove suggests that diverse microbial antigens can be presented.

### Effector functions of group 1 and group 2 CD1-restricted T cells

Group 1 and group 2 CD1-restricted T cells display antigen-specific cytotoxicity in the context of sterile cancer and infectious disease. Like group 1 CD1 autoreactive T cells, type I and type II NKT cells are capable of directly lysing tumor cells (100–103). Upon activation, type I NKT cells can produce both Th1 (IFN-γ, TNF-α) and Th2 (IL-4, IL-5, IL-13) cytokines, and this dual functionality enables them to play both pathogenic and immunoregulatory roles in various disease settings (104). Type I NKT cells were also shown to produce anti-inflammatory cytokines, such as IL-10 and TGF-β. Similar to type I NKT cells, type II NKT cells are also capable of producing multiple cytokines in response to polyclonal TCR stimulation, including IFN-γ, IL-4, GM-CSF, and IL-13 (105). These findings show that group 2 CD1-restricted NKT cells can produce a variety of Th1, Th2, and regulatory cytokines in response to different environmental cues.

Group 1 CD1-restricted T cells have also been shown to produce multiple cytokines in the context of infection, inflammation, and cancer. Autoreactive group 1 CD1-restricted T cell lines produce IFN-γ, TNF-α, IL-17, and IL-22 in response to self-antigenic stimulation (5). Both *in vitro* and *in vivo* assays demonstrated that group 1 CD1-restricted T cells produce IFN-γ and TNF-α upon encountering mycobacterial antigens (4, 65, 66). Unlike group 2 CD1-restricted T cells, most group 1 CD1-restricted T cells do not appear to produce Th2 and/or regulatory cytokines in response to antigenic stimulation. However, to date, all the studies examining group 1 CD1-restricted T cells do so in *Mtb* infection settings. Further experiments need to be conducted to ascertain whether group 1 CD1-restricted T cells produce Th2 and/or regulatory cytokines under other infectious or inflammatory conditions.

### Antigen-presenting cell–T cell interactions

**Figure 1:**
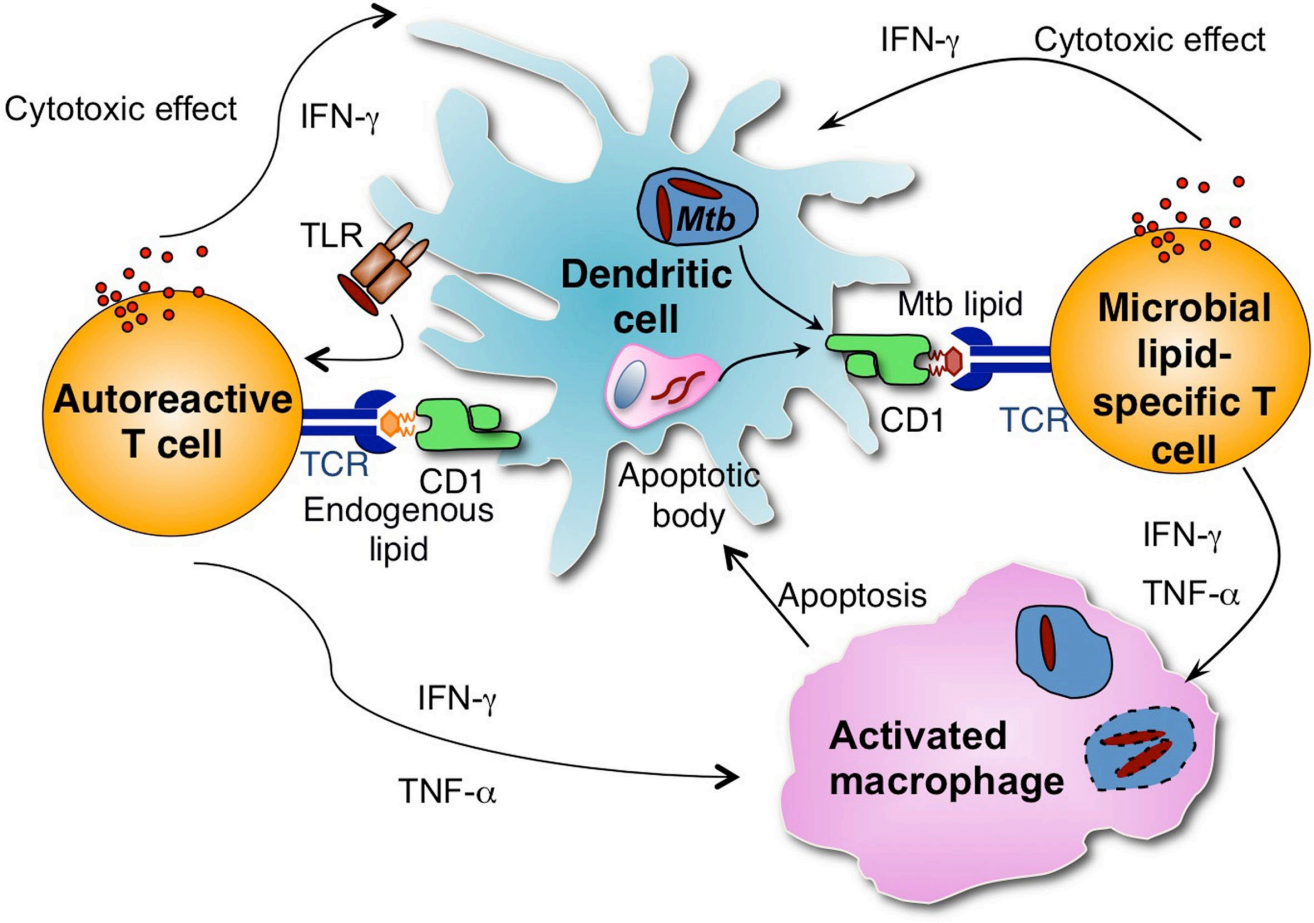
**Group 1 CD1-restricted T cells consist of autoreactive and microbial antigen-specific T cells**. Microbial lipid-specific T cells are activated by microbial lipids that are loaded on group 1 CD1 molecules. Autoreactive T cells are activated in combination by proinflammatory cytokines produced on TLR engagement and self-lipid antigens. During *Mtb* infection, group 1 CD1-expressing DCs can either be directly infected by *Mtb* or take up apoptotic vesicles from bystander infected cells and present *Mtb*-derived antigens to group 1 CD1-restricted *Mtb* lipid-specific T cells. Activated group 1 CD1-restricted T cells are cytotoxic and can directly lyse *Mtb*-infected cells. Activated group 1 CD1-restricted T cells also produce IFN-γ and TNF-α, which can activate infected macrophages and help to control *Mtb* growth.

Although all APCs expressing CD1d have the potential to present lipid antigens to NKT cells, studies depleting specific types of APCs have revealed that APC subsets differ in their capacity to prime type I NKT cells with lipid antigens (106). A recent study demonstrated that CD8α^+^ DEC-205^+^ dendritic cells were the main cell type responsible for capturing and presenting multiple forms of α-GalCer and stimulated type I NKT cell responses *in vivo* (106). However, it is unclear which APC subset is responsible for presenting *Mtb* antigens to group 1 CD1-restricted T cells *in vivo*. Macrophages are the primary host cells for *Mtb* but do not express group 1 CD1 molecules. It was demonstrated that group 1 CD1-expressing DCs take up apoptotic vesicles from bystander infected cells and presented them to human CD8^+^ T cells in an *in vitro* co-culture experiment (107). Thus, group 1 CD1-expressing DCs may similarly cross-present *Mtb* lipid antigens to T cells during infection (Figure 1).

Most of the studies involving CD1d have been performed in murine models. However, as humans possess both group 1 CD1 and group 2 CD1 molecules, it is possible that presence of group 1 CD1 molecules may affect CD1d antigen presentation. While there is some specialization in the antigen presentation functions of CD1 molecules, whereby they sample lipids from different intracellular compartments, sulfatide, a glycosphingolipid, is capable of promiscuously binding to CD1a, CD1b, CD1c, and CD1d (59). As CD1c tends to be co-expressed with CD1d on blood dendritic cells and a fraction of B cells, a recent study evaluated how the presence of CD1c influences the activation of CD1d-restricted iNKT cells (108). This study demonstrated that CD1c is able to present α-GalCer as a weak agonist to human iNKT cells, and the presence of CD1c enhances α-GalCer-dependent activation of iNKT cells by CD1d (108).

### Role of group 1 and group 2 CD1-restricted T cells during infection

The studies described above support the likely role of group 1 CD1-restricted T cells in protective immunity against *Mtb* infection. However, the mechanisms by which they control *Mtb* infection remain to be elucidated. Aside from *Mtb*, the role of group 1 CD1-restricted T cells in other microbial infection has not been addressed. In contrast, the role of CD1d-restricted iNKT cells in host defense against various pathogens has been extensively studied (109). Studies examining iNKT cell dynamics in mice infected intravenously with BCG showed an early expansion of iNKT cells in the lung with a peak at day 8 after infection, followed by a subsequent decline likely due to programed cell death (110, 111). Other studies have suggested that iNKT cells may play a role in the formation of granulomas in response to mycobacterial cell wall components (112, 113). Furthermore, *in vitro* experiments have shown that iNKT cells possess the ability to kill *Mtb*-infected APCs through the production of GM-CSF (114). However, *Mtb* infection of CD1d^−/−^ or Jα18^−/−^ mice with *Mtb* resulted in no significant difference in mortality or bacterial burdens in the lung, suggesting that iNKT cells may play a redundant role in the control of *Mtb* (115–117).

## Conclusion

We highlight several recent studies that have contributed to our understanding of group 1 CD1 antigen presentation and the role group 1 CD1-restricted T cells play during *Mtb* infection. Further studies are needed to characterize *Mtb* lipid-specific T cell memory responses and evaluate whether *Mtb* lipid antigens can effectively be used in a subunit vaccine. Future studies also need to expand on the role of autoreactive group 1 CD1-restricted T cells in *Mtb* infections, as it is unknown whether they have similar activation kinetics as antigen-specific T cells and how they affect host immunity to infection. Moreover, it remains unclear whether group 1 CD1 molecules present lipids from other microbial species and whether they play a role in other inflammatory diseases, presenting fertile ground for future investigation.

## Conflict of Interest Statement

The authors declare that the research was conducted in the absence of any commercial or financial relationships that could be construed as a potential conflict of interest.
